# Vulnerable Narcissism and Celebrity Worship: The Mediating and Moderating Role of Commitment to Parasocial Relationships

**DOI:** 10.3390/bs16030333

**Published:** 2026-02-27

**Authors:** Lawrence Locker, Joshua L. Williams, Jeff Klibert

**Affiliations:** 1 Department of Psychology, Georgia Southern University, P.O. Box 8041, Statesboro, GA 30460, USA; jklibert@georgiasouthern.edu; 2 Department of Criminal Justice and Criminology, Georgia Southern University, P.O. Box 8105, Statesboro, GA 30460, USA

**Keywords:** celebrity worship, vulnerable narcissism, parasocial relationships

## Abstract

The current study further investigated the relationship between celebrity worship and vulnerable narcissism by examining the mediating role of commitment to parasocial relationships to a celebrity or media figure. Positive relationships among the variables emerged such that greater celebrity worship was associated with higher levels of vulnerable narcissism and commitment to parasocial relationships. The degree of commitment to parasocial relationships was also positively related to levels of vulnerable narcissism. Commitment to parasocial relationships also partially mediated the relationship between vulnerable narcissism and celebrity worship. These results suggest the importance of investigating the parasocial component of celebrity worship and support prior research concerning the connection between celebrity worship and vulnerable narcissism.

## 1. Introduction

Celebrity worship (i.e., excessive attachment to a celebrity) is an increasingly problematic phenomenon (McCutcheon & Aruguete, 2021). One way in which celebrity worship is conceptualized is through an absorption-addiction framework (McCutcheon et al., 2002). The absorption-addiction model assumes a continuum ranging from entertainment-social to borderline-pathological levels of celebrity attachment. The Celebrity Attitude Scale (CAS) was developed as a metric by which to assess an individual’s point on this continuum from the relatively benign and less maladaptive entertainment-social level to the more maladaptive Intense-Personal and Borderline-Pathological levels (McCutcheon et al., 2002; Maltby et al., 2004). The initial interest in a celebrity is assumed to begin at the entertainment-social level in which the interest in the celebrity serves as a means of making social connections among others interested in the celebrity, and as a means of entertainment. However, for individuals who, for example, may have psychological distress or a poorly developed sense of self, absorption in the celebrity may increasingly develop in intensity as a means of coping and consequently, one may progress to an intense-personal level involving, for example more intrusive and even unwanted thoughts and a more unrealistic sense of connection to the celebrity (e.g., perceiving a celebrity as one’s soulmate). A few individuals progress to the most extreme and maladaptive borderline-pathological level at which point one may present behaviors similar to addiction. The borderline-pathological level is characterized, for example, by willingness to engage in extreme behaviors such as spending large amounts of money on a trivial item (i.e., a napkin used by a celebrity) or willingness to engage in illegal behavior if a favored celebrity requested it. In sum, celebrity worship can, in some individuals, serve as an increasingly maladaptive means of coping, whereby an individual becomes increasingly absorbed in and addicted to this attachment, leading to increasingly maladaptive, and in some cases, extreme behaviors (e.g., stalking, McCutcheon et al., 2002; Maltby et al., 2004; Wong et al., 2023). Over the last two decades, there is a growing body of evidence supporting the absorption-addiction framework; higher levels of celebrity worship are linked to a host of negative outcomes, including poor mental health, materialism, and narcissism (Davis et al., 2025; Greenwood et al., 2018; Maltby et al., 2004). Given the negative impact of celebrity worship, it is important to identify risk factors and models that explain how celebrity worship develops. Given the broad range of negative psychological correlates (see Brooks, 2018 for review), the study of celebrity worship has been shown to be relevant to a number of domains of study such as personality (i.e., narcissism), mental health, addiction, criminality, and problematic use of technology (e.g., Greenwood et al., 2018; Locker & Williams, 2025; Griffiths, 2024; Sheridan et al., 2007; Wei & Huang, 2026; Wong et al., 2023; Zsila et al., 2018, 2020).

### Celebrity Worship and Narcissism

There is a body of work demonstrating that extreme celebrity worship is associated with several negative psychological factors (Brooks, 2018). One correlate of celebrity worship is narcissism, particularly vulnerable narcissism (Davis et al., 2025; Greenwood et al., 2018). Narcissism is a complex, dynamic construct and a difficult one to define as the term encompasses, for example, motivation based on extreme self-focus, a phase of development and personality disorder (Krizan & Herlache, 2018). Krizan and Herlache in their model defined a core feature of narcissism as entitlement and self-importance that involves a perspective of one’s own needs or wants to be more important than others’, with a more extreme sense of self-importance or being more deserving than others, a core feature on a continuum encompassing two subtypes. Because of variations in expression and experience, researchers often categorize narcissism into two subtypes (vulnerable and grandiose). While these subtypes overlap in terms of a strong focus on the self, antagonistic interpersonal interactions, and arrogance, there are unique elements to each (Miller et al., 2011; Krizan & Herlache, 2018; Wink, 1991). For example, from a personality perspective, vulnerable narcissism is connected to higher levels of distress, anxiety, and neuroticism, whereas the grandiose narcissism is linked to lower levels of negative affect and higher levels of extraversion (Miller et al., 2011). Miller and colleagues described the overlap and differences among the two subtypes as grandiose narcissism being characterized by aggression and dominance whereas vulnerable narcissism being characterized by a sense of grandiosity that is both insecure and masks feelings of negative emotions and perceptions of inadequacy or incompetence (Miller et al., 2011). Although a full description of documented differences among the narcissistic subtypes is beyond the scope of the present paper, researchers have documented a number of distinguishing correlates. For example, grandiose narcissism has been found to be associated with extraversion whereas vulnerable narcissism appears to be associated with higher neuroticism, self-consciousness, and anxiety (Miller et al., 2011). Miller et al. (2021) described differences among the two subtypes in terms of, for example, greater psychological distress and by extension more interpersonal difficulties associated with vulnerable narcissism, whereas grandiose narcissism is associated with less interpersonal difficulty and more proactive (i.e., agentic extraversion) rather than reactive in nature. In a study of the subtypes among individuals who had engaged in and been convicted of criminal behavior, grandiose narcissism was found, for example, to be positively associated with levels of self-control and negatively with criminal behavior, whereas the opposite was found for individuals higher in vulnerable narcissism (Bogaerts et al., 2021). In an examination of bulimia nervosa in undergraduate women, symptoms of bulimia were found to be significantly associated with vulnerable, but not grandiose narcissism, which the authors had expected to find based on the negative emotionality (e.g., anxiety and shame) related to both bulimia nervosa and vulnerable narcissism (Maples et al., 2011). While the evidence for a relationship between grandiose narcissism and celebrity worship is mixed (Ashe et al., 2005), there are consistent, positive associations between vulnerable narcissism and celebrity worship (Davis et al., 2025; Greenwood et al., 2018; Williams et al., 2025). Ashe et al. (2005) first examined the relationship between grandiose narcissism and celebrity worship in two samples, one from the United States (US) and one from the United Kingdom (UK). Their results revealed a positive relationship between levels of narcissism and celebrity worship although the connection was more robust in the UK than the US sample. A later study by Greenwood et al. (2018) extended this research by examining both subtypes of narcissism in relation to celebrity worship. They failed to replicate the pattern found by Ashe et al. (2005) for the grandiose subtype but did find a positive correlation between celebrity worship and the vulnerable subtype. More recent studies replicated the pattern observed by Greenwood and colleagues of a significant relationship between celebrity worship and the vulnerable, but not grandiose subtype of narcissism (Davis et al., 2025; Locker & Williams, 2025; Locker et al., 2025; Williams et al., 2025). Conceptually, the pattern concerning the two subtypes of narcissism and celebrity worship align with an absorption-addiction framework of extreme celebrity attachment as proposed by McCutcheon and colleagues (McCutcheon et al., 2002) as the vulnerable subtype is more highly associated with poorer well-being (Miller et al., 2011). For instance, researchers highlight parasocial relationships (i.e., one-sided, non-reciprocated relationship) as a potential explanation concerning how vulnerable narcissism traits possibly contribute to celebrity worship (Greenwood et al., 2018). This theoretical pattern fits well with the absorption-addiction framework, whereby the nature of parasocial relationships contributes to how celebrity worshipers compensate for unfulfilling lives and obtain a sense of purpose. While there are clear connections between vulnerable narcissism and celebrity worship, the mechanisms underlying this relationship have yet to be fully explored. For example, there is little research that has explicitly examined potential mediators or moderators of this relationship to better understand the specific psychological mechanisms underlying the connection between vulnerable narcissism and celebrity worship (e.g., social difficulties, need for connection to others, poorer self-concept). A recent study by Locker and Williams (2025) did attempt to examine negative emotionality as a potential moderator of this relationship. Their results replicated the pattern of a connection between celebrity worship and the vulnerable, but not grandiose, subtype of narcissism. Congruent with theoretical differences among the subtypes, negative emotionality was associated with the vulnerable, but not the grandiose subtype. However, their results did not find that negative emotionality moderated the relationship between celebrity worship and vulnerable narcissism. Therefore, there remains a gap in the literature regarding the specific mechanisms and psychological variables that could account for this connection.

The current study focused on the parasocial component of celebrity worship (e.g., Zsila et al., 2025). As noted above, parasocial relationships are defined as one-sided connections to an individual (Horton & Wohl, 1956) such that, for example, one could be emotionally involved in another individual such as a favored celebrity, perhaps devoting considerable thought to learning about the celebrity or feeling a strong emotion connection, but given that the celebrity may not even be aware of the fan’s existence, the connection is one-sided. Since Horton and Wohl’s (1956) paper, the topic of parasocial connections has received considerable attention, with one review finding that a majority of studies have been published relatively recently (i.e., 2016–2020) in comparison to the 60 years prior to that period, with interest in the topic including areas or domains such as communication science, media and entertainment studies, advertising, and health and political communication among others (Schramm et al., 2024). There also has been an increased focus on social/new media contexts in recent years as opposed to the greater focus on television/film contexts in previous periods (Schramm et al., 2024; Liebers & Schramm, 2019). The complexity of parasocial connections is yet to be fully understood, being associated with negative outcomes such as maladaptive celebrity attachment and its negative correlates (Brooks, 2018) but also positive outcomes relating to factors such as emotion regulation, meeting emotional needs, feelings about the self, and well-being (e.g., Koroglu et al., 2025; Lotun et al., 2024; Paravati et al., 2024; Stein et al., 2024; see also Hoffner & Bond, 2022 for a review of parasocial connections and outcomes in the context of well-being). Although not exhaustive, the complex nature of parasocial connections (e.g., relations with both positive and negative psychological outcomes) in different contexts discussed above illustrates the importance of further consideration of the role that it may play in the connection between celebrity worship and the negative psychological outcomes reported in the literature, such as vulnerable narcissism considered in the current study (e.g., Greenwood et al., 2018; Locker et al., 2025; see also Brooks, 2018).

Linkages among celebrity worship, parasocial relationships, and narcissism are evident within the literature. Engagement in parasocial relationships is positively related to celebrity worship (Williams et al., 2024; Zsila et al., 2025). Recent empirical evidence supporting the notion of the construct of celebrity worship includes a strong parasocial component has been demonstrated using the Multidimensional Measure of Parasocial Relationships (MMPR) that measures parasocial commitment in terms of attitudes along cognitive, affective, behavioral, and decisional dimensions (Garcia et al., 2022). A robust positive correlation has been observed between the MMPR and celebrity worship as measured by the CAS, supporting the importance of the parasocial connection in absorption in a favored celebrity (Williams et al., 2024; Zsila et al., 2025). In addition, narcissistic behavior has been shown to be positively related to elements of parasocial relationships, including increased guidance seeking, desire for face-to-face contact, and feelings of intimacy toward a fictional celebrity character, for example (Brodie & Ingram, 2021). Vulnerable narcissism, in particular, has emerged in recent research to be consistently and significantly predictive of engaging parasocial relationships, especially celebrity worship. Theoretically, such findings fit well within the Absorption-Addiction framework such that establishing a parasocial relationship may be a maladaptive approach to coping with poor self-esteem, anxiety, social difficulties, negative affect, among others, all of which have been more associated with vulnerable narcissism (Greenwood et al., 2018; Locker & Williams, 2025; Locker et al., 2025; McCutcheon et al., 2002; Williams et al., 2025). Given these patterns of connection and the need to explore the underlying mechanisms of the absorption-addiction framework more fully, the aim of the current study was to determine whether parasocial relationships indirectly account for covariance in the vulnerable narcissism-celebrity worship relationship. Additionally, prior research suggests a relationship between celebrity worship, age, and gender (e.g., Zsila et al., 2021), and therefore we included these two demographic variables as covariates.

In terms of hypotheses, we expected positive relationships among celebrity worship, parasocial relationships, and vulnerable narcissism. We also expected parasocial relationships to mediate the relation between vulnerable narcissism and celebrity worship. We also conducted an exploratory moderation test to determine if the connection between vulnerable narcissism and celebrity worship differed as a function of parasocial commitment. If findings hold to expectations, our model may offer unique insights into how problematic personality styles and interpersonal patterns of behavior may contribute to celebrity worship. 

## 2. Materials and Methods

### 2.1. Participants

Participants were 293 undergraduate students at a southeastern university in the United States who participated for course credit or extra credit. Sixty-five participants were excluded either for failing an attention check question or not completing the study. Of the remaining 228, participants that did not provide a response to the age or biological sex demographic questions were not included in analyses. Of the 218 participants included in the analyses, 161 were women, 57 were men. The mean age of the sample was 21.73 years (*SD* = 5.46). One hundred sixty-one participants identified as European or White (73.9%), 48 as African American or Black (22%), 18 as Hispanic, Latino or Spanish origin (8.3%), seven as Asian (3.2%), two as Middle Eastern or North African (0.9%), six as American Indian or Alaska Native (2.8%), and three preferred not to respond (1.4%)^1^.

### 2.2. Materials and Procedure

Approval from the Institutional Review Board (IRB) was obtained. Following IRB approval, undergraduate students were recruited either through course instructors or through the online research management system (SONA) in psychology. The study was conducted online via Qualtrics, and participants completed the study on their own electronic devices. Participants reviewed the informed consent and either chose to continue with the study or exit the system. Next, participants provided their favorite celebrity or social media influencer and were instructed to consider this individual when responding to two measures (CAS and MMPR). Participants completed the following measures in a randomized order followed by the demographic questions.

The Celebrity Attitude Scale (CAS; McCutcheon et al., 2002) measured celebrity worship. This measure includes 23-items to which participants respond on a 1 (strongly disagree) to 5 (strongly agree) rating scale. Higher values represent stronger levels of celebrity worship. Sample items of this measure are “I consider my favorite celebrity to be my soul mate,” and “I love to talk with others who admire my favorite celebrity.”

The *CAS* has demonstrated solid internal consistency over several decades (Cronbach’s α = 0.84 to 0.94; Brooks, 2018). The CAS demonstrated excellent internal consistency (α = 0.94) in the current study.

The Maladaptive Covert Narcissism Scale (MCNS; Cheek et al., 2013*)* assessed vulnerable narcissism. The MCNS comprises 23 items rated on a 1 (very uncharacteristic or untrue, strongly disagree) to 5 (very characteristic or true, strongly agree) rating scale with higher scores reflecting greater levels of vulnerable narcissism. Sample items of this measure are “I tend to feel humiliated when criticized,” and “I resent others who have what I lack.” The *MCNS* has demonstrated solid internal consistency in past administrations (Cronbach’s α = 0.83; Cheek et al., 2013). Within the current study, the *MCNS* demonstrated excellent internal consistency (α = 0.92).

The Multidimensional Measure of Parasocial Relationships (MMPR; Garcia et al., 2022) measured commitment to parasocial relationships. This scale comprises 18 items to which participants respond on a 1 (Totally Disagree) to 4 (Totally Agree) rating scale with higher scores representing higher commitment to parasocial relationships. Sample items of this measure are “I experience a feeling of connectedness with the media figure through his/her posts on social media,” and “I think that the media figure represents values that are important to me.” The measure has demonstrated solid internal consistency (Cronbach’s α = 0.85; Garcia et al., 2022). Within the current study, the measure demonstrated solid internal consistency (α = 0.85).

## 3. Results

Bivariate correlations were conducted to examine the strength and direction of the relationships among the study’s main variables. Results revealed positive relationships among celebrity worship (*CAS*), vulnerable narcissism (*MCNS*) and parasocial commitment (*MMPR*) scores. Biological sex did not correlate significantly with the primary study measures, but age was negatively and significantly correlated with celebrity worship and vulnerable narcissism, but not parasocial commitment (see Table 1).

**Figure 1:**
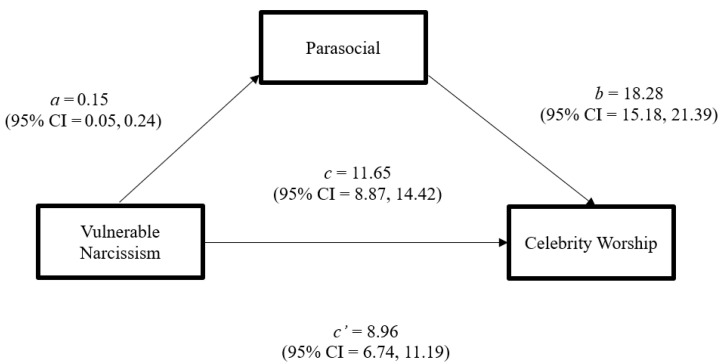
Unstandardized Coefficients for the Indirect and Direct Effect of the Mediation Model. Notation: For the a and b pathways, coefficients (b) and 95% confidence intervals (CI) are reported. Direct (c’) and total effect (c) coefficients and CIs are also reported.

Indirect and direct effects among the study’s variables were analyzed through a mediation model using the PROCESS macro (Model 4; Hayes, 2022) with age and biological sex included as covariates. Within the model, celebrity worship was regressed upon vulnerable narcissism (focal predictor), commitment to parasocial relationships (mediator) age, and biological sex. Results indicated the model (see Figure 1) accounted for 55% of the variance in celebrity worship scores, *F*(4, 213) = 64.10, *p* < 0.001. The indirect effect was estimated using a bootstrap analysis of 10,000 samples (CI = 95%) with a random seed of 12,345 for reproducibility of the indirect effect estimates. Results revealed partial mediation with both the total effect (*p* < 0.0001) and the direct effect (*p* < 0.0001) being statistically significant. The indirect effect, b = 2.68 SE = 0.94, 95% CI [0.85, 4.53], was also significant, indicating that commitment to parasocial relationships explained some of the covariance between vulnerable narcissism and celebrity worship. Age and biological sex were not significant predictors in the model.

**Figure 2:**
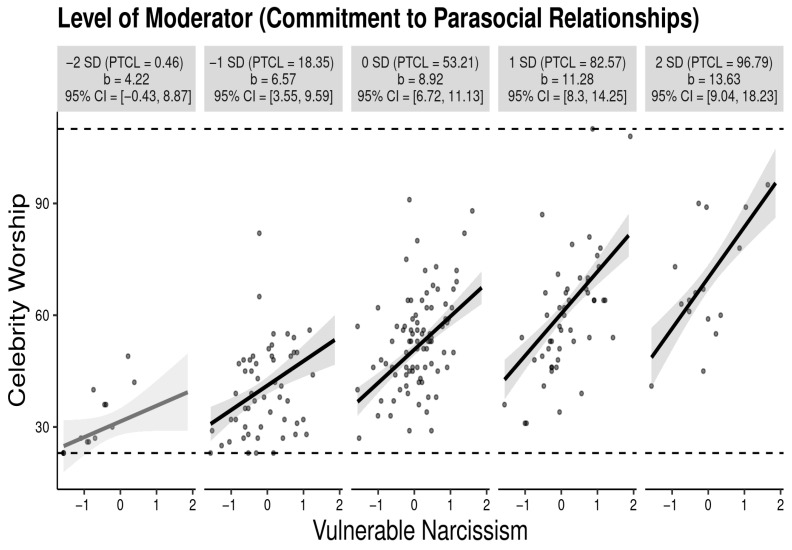
The simple slopes graph depicts the positive relationship between vulnerable narcissism and celebrity at different levels of commitment to parasocial relationships. The far-left panel depicts the relationship at extremely low levels of the commitment to parasocial relationships, the next panel depicts the relationship at moderately low levels of commitment to parasocial relationships, the mid panel depicts the relationship at moderate levels of commitment to parasocial relationships, the next panel depicts the relationship at moderately high levels of commitment to parasocial relationships, and the far-right panel depicts the relationship at extremely high levels of commitment to parasocial relationships. The relationship between vulnerable narcissism and celebrity is significant at every level of commitment to parasocial relationships with one exception. The relationship between vulnerable narcissism and celebrity is non-significant at extremely low levels of commitment to parasocial relationships.

**Figure 3:**
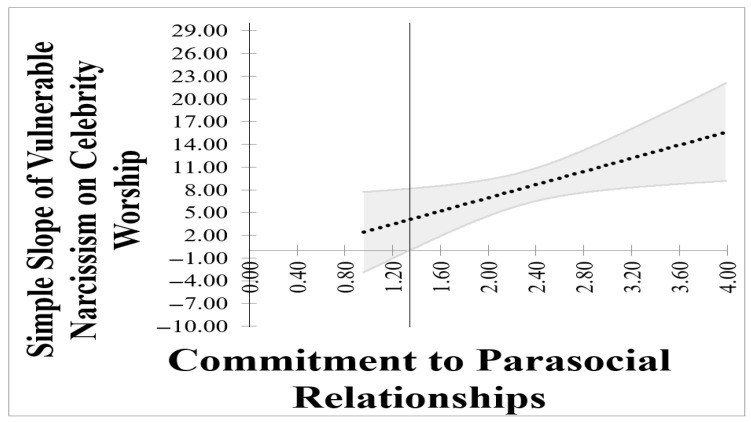
The (unstandardized) magnitude of the relationship between vulnerable narcissism and celebrity worship as a function of commitment to parasocial relationships. The gray curved lines represent the upper and lower confidence bounds for estimating the relationship between vulnerable narcissism and celebrity worship. The vertical lines mark the regions of significance. The association between vulnerable narcissism and celebrity worship is projected to cease when the average among all commitment to parasocial relationship items are less than 1.34.

As an exploratory exercise, we ran a simple moderated model with covariates (age, sex) to evaluate whether the relationship between vulnerable narcissism and celebrity worship varied as a function of commitment to parasocial relationships. Regression statistics are presented in Table 2. The main and interaction effects account for 55.7% of the variance in celebrity worship, *F*(5, 212) = 53.34, *p* < 0.01. Individually, the main effects for vulnerable narcissism and commitment to parasocial relationships were significant. Similarly, the interaction between vulnerable narcissism and commitment to parasocial relationships was significant, indicating a significant moderation effect. Because a significant moderation effect was detected, it was important to probe the interaction. We probed the interaction effect using a simple slopes analysis (interactive utility tool; McCabe et al., 2018). Because slopes use arbitrary labels to evaluate interactions, we also analyzed Johnson-Neyman data using a Johnson-Neyman tool (CAHOST) Johnson-Beyman excel workbook (Carden et al., 2017). The simple slopes analysis is presented in Figure 2 and the Johnson-Neyman data is presented in Figure 3. In the far-right panel of Figure 2 (extremely high levels of commitment to parasocial relationships), the relationship between vulnerable narcissism and celebrity is very strong. Yet, in the far-left panel of the figure (extremely low levels of commitment to parasocial relationships, the relationship between vulnerable narcissism is non-significant. Within Figure 3, the dark vertical line represents the exact point on commitment to parasocial relationships by which the relationship between vulnerable narcissism and celebrity worship becomes non-significant. The figure indicates that the relationship between vulnerable narcissism and celebrity worship is non-significant when the average scores on the *MMPR* is 1.34. Overall, 8.26% of the sample reported a non-significant relationship between vulnerable narcissism and celebrity worship.

## 4. Discussion

The current study evaluated more nuanced connections between vulnerable narcissism and celebrity worship by exploring the mediating role of commitment to parasocial relationships. Consistent with our expectations, there was a positive relationship between vulnerable narcissism and celebrity worship. Additionally, stronger commitments to parasocial connections were related to greater engagement in celebrity worship. In sum, these findings are consistent with previous literature (Davis et al., 2025; Greenwood et al., 2018) and confirm specific personality and interpersonal styles meet basic methodological criteria for risk factors of celebrity worship.

Our findings also offer some clarity regarding the nuances inherent within the relationship between vulnerable narcissism and celebrity worship. Specifically, engaging in parasocial relationships is useful in explaining (mediating) how vulnerable narcissism traits relate to higher levels of celebrity worship. The direct effects between vulnerable narcissism and celebrity worship were significant after the inclusion of parasocial relationships, suggesting a partially mediated effect. This finding was consistent with Greenwood et al.’s (2018) assertion that the parasocial nature of celebrity worship may be more appealing for the vulnerable than the grandiose subtype of narcissism. Furthermore, this finding integrates unique literature bases into a more defined model of celebrity worship (vulnerable narcissism → parasocial interpersonal styles → celebrity worship). The moderation analysis provided further insight into this relationship, revealing that the relationship between vulnerable narcissism and celebrity worship was moderated by level of parasocial commitment. Vulnerable narcissism was predictive at all but the lowest level of parasocial commitment and celebrity worship was highest at high levels of vulnerable narcissism and parasocial commitment. This finding supports Greenwood and colleagues’ argument that the parasocial nature of celebrity worship may be appealing to individuals higher in vulnerable narcissism and may be one explanatory factor in the apparent stronger connection of the vulnerable subtype of narcissism with celebrity worship observed in recent studies (Davis et al., 2025; Greenwood et al., 2018; Locker & Williams, 2025; Locker et al., 2025; Williams et al., 2025). At a theoretical level, these models imply that vulnerable narcissists are more likely to engage in parasocial relationships, which in turn may increase the risk for celebrity worship. However, any conclusions drawn from these findings must be situated within the limitations of the design, which was cross-sectional and correlational. In fact, mediation analyses are designed to evaluate causal relationships to better understand how chains of constructs bring forth a more complete understanding of ubiquitous and troublesome outcomes. Future longitudinal and experimental studies are required to verify the viability of the model in explaining celebrity worship outcomes. Specifically, future research should evaluate the strength and directional (vulnerable narcissism → parasocial interpersonal styles → celebrity worship) effects of our model. While our model was conceived from tenets of the absorption-addiction framework, it is quite possible these variables follow a different directional chain. Evaluating and estimating models of best fit through causal analysis will better clarify the nature of these relationships.

The current results also converge with an absorption-addiction view of maladaptive celebrity attachment, in that attraction to a celebrity may partially serve the purpose of coping whether in terms of sense of self or poorer well-being (Maltby et al., 2004), as would be expected to a greater degree with the vulnerable subtype of narcissism (as opposed to the grandiose subtype). Given the psychological distress and associated interpersonal difficulties connected with the vulnerable subtype, the parasocial element of celebrity worship would then be both appealing and possibly serve as a means of coping with the negative feelings, which would be in line with the absorption-addiction model (Greenwood et al., 2018; Maltby et al., 2004, Miller et al., 2021). In addition to shedding light on the recent findings concerning celebrity worship and its differential connection with the narcissistic subtypes, this study also illustrates the importance of examining the parasocial component of celebrity worship. Further exploring the role that the parasocial component plays in celebrity worship and its correlations with negative psychological factors beyond narcissism may be important given that not only is there evidence that celebrity worship has been on the rise, but with current technology, the opportunity for forming parasocial connections is undoubtably also on the rise (Garcia et al., 2022; McCutcheon & Aruguete, 2021).

### Limitations and Future Directions

A limitation of the current study is that the participants were limited to a convenience sample of undergraduate students who predominantly identified as White women. Future studies should examine the relationship between vulnerable narcissism and celebrity worship within a sample representing a broader portion of the population regarding age, sex, and ethnicity. Given that the connection to parasocial relationships only partially mediated the relationship between vulnerable narcissism and celebrity worship, and the complexity of vulnerable narcissism as a construct, investigating other mediators and moderators (e.g., anxiety) in this context would likely yield additional insights. Finally, the current study design limits the causal inferences that can be drawn from the mediation analysis. The psychological mechanisms underlying the relationship between vulnerable narcissism and celebrity worship, as well as those underlying parasocial commitment in relation to celebrity worship and vulnerable narcissism, are currently under explored. Future research should examine further the potential mediators/moderators of the relationships among all the variables in the current study, including but not limited to factors such as need for intimacy and deficiencies in identity formation. Future research should also examine a broader range of potential covariates than what was included in the current study. Despite these limitations, the current study extends work in celebrity worship by examining the role commitment to parasocial relationships plays in the connection between vulnerable narcissism and maladaptive celebrity worship. Examination of such factors might provide better insight into why some individuals progress from simply enjoying or admiring the work of a celebrity to a maladaptive level of attachment or absorption.

## Figures and Tables

**Table 1:** Descriptive Statistics and Bivariate Correlations.

Variable	Mean	SD	1	2	3	4	5
CAS	51.13	16.84	--				
MCNS	2.57	0.72	0.50 **	--			
MMPR	2.47	0.51	0.63 **	0.21 **	--		
AGE	21.73	5.46	−0.14 *	−0.18 **	−0.06	--	
SEX	--	--	−0.03	0.06	0.03	−0.09	--

Note: ** *p* < 0.01, * *p* < 0.05; CAS = Celebrity Attitude Scale; MCNS = Maladaptive Covert. Narcissism Scale; MMPR *=* Multidimensional Measure of Parasocial Relationships.

**Table 2:** Regression Values for the Moderating Effect of Commitment to Parasocial Relationships on the Relationship between Vulnerable Narcissism and Celebrity Worship.

Variables	*b*	*SE*	*t*	95% LLCI	95% ULCI
Main Effects					
Vulnerable Narcissism	8.92	1.12	7.98 **	6.72	11.13
Commitment to Parasocial Relationships	19.08	1.6	11.95 **	15.93	22.23
Interaction Effect					
Vulnerable Narcissism x Commitment to Parasocial Relationships	4.66	2.04	2.29 **	0.64	8.68
Covariate Effects					
Age	−0.15	0.14	−1.03	−0.43	0.14
Sex	−3.23	1.76	−1.83	−6.71	0.24

Note: *b* = unstandardized regression coefficient; *SE* = standard error; *LLCI* = lower-level confidence interval; *ULCI* = upper-level confidence interval. ** *p* < 0.01.

## Data Availability

The original data presented in the study are openly available in FigShare at https://doi.org/10.6084/m9.figshare.31242475.

## Note

1 Percentages may exceed 100% as participants were allowed to select more than one option.

## Abbreviations

The following abbreviations are used in this manuscript:

CAS	Celebrity Attitude Scale
IRB	Institutional Review Board
MMPR	Multidimensional Measure of Parasocial Relationships
MCNS	Maladaptive Covert Narcissism Scale
